# Genotypic and phenotypic spectrum of maple syrup urine disease in Zhejiang of China

**DOI:** 10.1093/qjmed/hcae104

**Published:** 2024-06-05

**Authors:** X Yang, R Yang, T Zhang, D J Tan, R Pan, Z Chen, D Wu, C Chen, Y Xu, L Zhang, X Li, Q Shu, L Hu

**Affiliations:** Department of Genetics and Metabolism, Children’s Hospital of Zhejiang University, School of Medicine, National Clinical Research Center for Child Health, Hangzhou, China; Department of Genetics and Metabolism, Children’s Hospital of Zhejiang University, School of Medicine, National Clinical Research Center for Child Health, Hangzhou, China; Department of Genetics and Metabolism, Children’s Hospital of Zhejiang University, School of Medicine, National Clinical Research Center for Child Health, Hangzhou, China; Department of Genetics and Metabolism, Children’s Hospital of Zhejiang University, School of Medicine, National Clinical Research Center for Child Health, Hangzhou, China; Department of Genetics and Metabolism, Children’s Hospital of Zhejiang University, School of Medicine, National Clinical Research Center for Child Health, Hangzhou, China; Department of Genetics and Metabolism, Children’s Hospital of Zhejiang University, School of Medicine, National Clinical Research Center for Child Health, Hangzhou, China; Department of Genetics and Metabolism, Children’s Hospital of Zhejiang University, School of Medicine, National Clinical Research Center for Child Health, Hangzhou, China; Department of Genetics and Metabolism, Children’s Hospital of Zhejiang University, School of Medicine, National Clinical Research Center for Child Health, Hangzhou, China; Department of Genetics and Metabolism, Children’s Hospital of Zhejiang University, School of Medicine, National Clinical Research Center for Child Health, Hangzhou, China; Department of Radiology, Children’s Hospital of Zhejiang University, School of Medicine, National Clinical Research Center for Child Health, Hangzhou, China; Department of Genetics and Metabolism, Children’s Hospital of Zhejiang University, School of Medicine, National Clinical Research Center for Child Health, Hangzhou, China; Department of Genetics and Metabolism, Children’s Hospital of Zhejiang University, School of Medicine, National Clinical Research Center for Child Health, Hangzhou, China; Department of Genetics and Metabolism, Children’s Hospital of Zhejiang University, School of Medicine, National Clinical Research Center for Child Health, Hangzhou, China

## Abstract

**Background:**

Maple syrup urine disease (MSUD) is an autosomal recessive metabolic disorder originating from defects in the branched-chain α-ketoacid dehydrogenase (BCKDH) complex encoded by *BCKDHA*, *BCKDHB* and *DBT*. This condition presents a spectrum of symptoms and potentially fatal outcomes. Although numerous mutations in the BCKDH complex genes associated with MSUD have been identified, the relationship between specific genotypes remains to be fully elucidated.

**Aim:**

Our objective was to predict the pathogenicity of these genetic mutations and establish potential links between genotypic alterations and the clinical phenotypes of MSUD.

**Design:**

Retrospective population-based cohort.

**Methods:**

We analyzed 20 MSUD patients from the Children’s Hospital at Zhejiang University School of Medicine (Hangzhou, China), recorded from January 2010 to December 2023. Patients’ blood samples were collected by heel-stick through neonatal screening, and amino acid profiles were measured by tandem mass spectrometry. *In silico* methods were employed to assess the pathogenicity, stability and biophysical properties. Various computation tools were utilized for assessment, namely PredictSNP, MAGPIE, iStable, Align GVGD, ConSurf and SNP effect.

**Results:**

We detected 25 distinct mutations, including 12 novel mutations. The *BCKDHB* gene was the most commonly affected (53.3%) compared to the *BCKDHA* gene (20.0%) and *DBT* gene (26.7%). *In silico* webservers predicted all novel mutations were disease-causing.

**Conclusions:**

This study highlights the genetic complexity of MSUD and underscores the importance of early detection and intervention. Integrating neonatal screening with advanced sequencing methodologies is pivotal in ensuring precise diagnosis and effective management of MSUD, thereby significantly improving the prognosis for individuals afflicted with this condition.

## Graphical abstract

**Figure:**
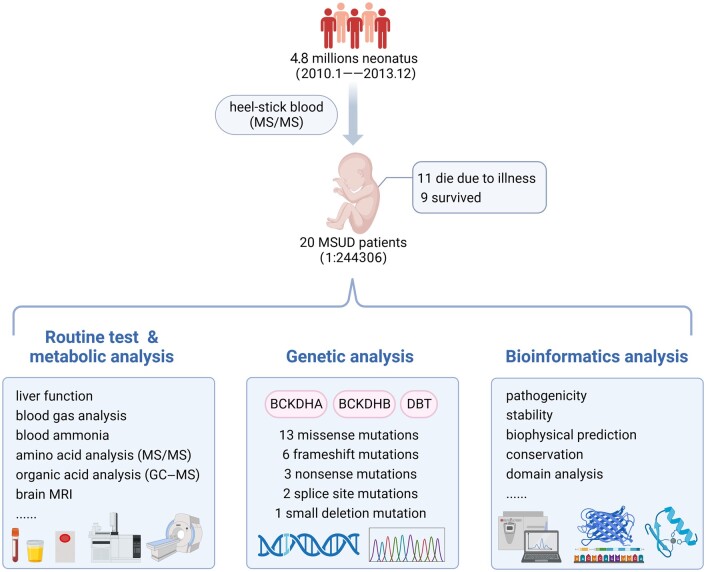

## Introduction

Maple syrup urine disease (MSUD, OMIM #248600) is a rare autosomal recessive disease with a prevalence of 1 in 150 000 among pediatric populations. It is hallmarked by the triad of encephalopathy, feeding difficulties and a distinctive odor of maple syrup in the urine.^1^^,^^2^ The fundamental mechanism underlying MSUD includes perturbations within the branched-chain α-ketoacid dehydrogenase (BCKAD) complex assembly, culminating in the aberrant accumulation of branched-chain amino acids (BCAAs) that induces toxicity within the skeletal muscle and the brain.^3^^,^^4^ Specifically, leucine, a major component of the BCAA class, is shown to directly influence subcortical grey matter by disrupting cerebral water homeostasis and augmenting the production of reactive oxygen species (ROS).^5^^,^^6^ Furthermore, α-ketoisocaproic acid, the intermediate of leucine metabolism, is also suggested to be neurotoxic.^1^^,^^7^^,^^8^

The diagnosis of MSUD is primarily established by quantifying the elevated levels of BCAA and the identification of alloisoleucine as a diagnostic biomarker.^2^^,^^9^ Research has demonstrated a strong correlation between mutations in the *DBT*, *BCKDHA* and *BCKDHB* genes and the onset of MSUD.^10–12^ However, a significant gap remains in comprehending the precise mechanisms underpinning these mutations. Next-generation sequencing (NGS) has emerged as a powerful tool for probing genomic alterations with high throughput.^13^^,^^14^ When combined with advanced bioinformatics, NGS data undergoes tertiary analysis,^15^ annotating functional impacts,^15^ assessing variation effects^16^ and predicting encoded proteins' biophysical attributes,^17^ stability^18^ and pathogenic potential.^19^ This integrated approach offers deep insights into molecular pathways, potentially elucidating disease mechanisms and aiding prognosis.

In the current study, we identified a cohort of 20 patients diagnosed with MSUD in Zhejiang of China. These patients underwent extensive metabolic profiling to delineate their clinical phenotypic profiles. Following this, we conducted a comprehensive NGS analysis to unravel genetic mutations, with a particular focus on individual SNPs.^20^^,^^21^ We then employed an array of advanced bioinformatics tools to elucidate the biophysical characteristics of the proteins expressed from these genetic variations.^18^^,^^22–24^ Our primary objective was to predict the pathogenicity of these genetic mutations and establish potential links between genotypic alterations and the clinical phenotypes of MSUD (Graphical Abstract). This holistic approach holds the potential to yield invaluable insights to monitor disease progression and anticipate its trajectories in follow-up assessments.

## Materials and methods

### Study population and sample collection

From January 2010 to December 2023, Children's Hospital at Zhejiang University School of Medicine (Zhejiang, China) gathered data from 20 patients suffering from MSUD. These patients were identified through a newborn screening program that covered 4.8 million Chinese newborns, revealing a prevalence rate of 1 in 244306. The study documented and examined their clinical profiles, biochemical information and long-term outcomes. The research received ethical clearance from the Ethical Committee of the Children's Hospital Zhejiang University School of Medicine under the approval number 2022-IRB-254. Written consent for the use of samples and publication of health information was provided by the guardians of all participating patients.

### Routine tests and metabolic analysis

The blood samples were collected by heel-stick and spotted on Whatman 903 filter paper for neonates 48 h to 7 days after birth. Tandem mass spectrometry (MS/MS) measured amino acid and acylcarnitine profiles with the NeoBase Non-derivatized MSMS Kit (PerkinElmer, Finland). Liver function, glucose, ammonia, lactic acid levels and blood gas compositions were monitored through routine physical examination and biochemical laboratory tests.

### Statistical analyses

SPSS 23.0 software was performed for statistical analysis and figure drawing. Continuous variables were described as mean ± standard deviation (SD). The Shapiro–Wilk normality test was used to determine whether the continuous variable is normally distributed or not. The Student’s *t*-test was used for comparing continuous variables with a normal distribution, while Kruskal–Wallis test was used to compare variables those were not normally distributed. The difference between two groups was compared by chi-square test. *P* values of less than 0.05 were considered as statistically significant.

### Targeted next-generation sequencing and mutational analyses

Peripheral blood leukocytes were used to extract genomic DNA from both the subjects and their parents, employing the QIAamp DNA Blood Mini Kit (Qiagen, Germany). A targeted approach to next-generation sequencing was applied, utilizing a hereditary metabolic disease panel that encompasses 306 genes, ensuring coverage of all coding regions and adjacent intronic sequences. Following a purification and preparation process, the samples were indexed for Illumina platforms using the TruePrepTM Index Kit V2 (Vazyme). Quantification of the sequencing libraries preceded their sequencing on an MGI DNBSEQ-T7 platform using the DNBSEQ-T7RS Reagent Kit v2, FCL PE150 (MGI, Shenzhen, China).

The sequencing output, post-cleaning, was aligned to the hg19 reference genome. SNP and InDel identification were performed, and annotations were provided *via* ANNOVAR software, referencing databases like 1000 Genomes, gnomAD, ClinVar and HGMD. These variants were evaluated and categorized according to the standards set by the American College of Medical Genetics and Genomics (ACMG). Additionally, co-segregation analysis was conducted through Sanger sequencing to confirm patterns.

### Pathogenicity, stability and biophysical prediction

*In silico* methods were employed to assess pathogenicity, stability and biophysical properties. Various computation tools were utilized for this assessment, namely PredictSNP, iStable and Align GVGD. Notably, PredictSNP serves as a consensus classifier by integrating multiple predictive algorithms such as MAPP, PhD-SNP, PolyPhen1, PolyPhen2, SIFT, SNAP, PANTHER and nsSNP analyzer.^21^ These tools offer valuable details about the nature of mutations, distinguishing between deleterious and neutral variants. The output of Multimodal Annotation Generated Pathogenic Impact Evaluator (MAGPIE) analyzer ranges from 0 to 1 (0 indicates a benign mutation, while 1 indicates a highly pathogenic mutation).^25^ iStable utilizes an SVM (support vector machine) to calculate the impact of an SNP (single nucleotide polymorphism) on protein stability. By combining predictions from I-Mutant 2.0, MUpro and iStable,^18^ integration enhances the predictive power, surpassing that of individual tools. Align GVGD merges protein multiple sequence alignment (MSA) with the biophysical properties of amino acids. It estimates the impact of non-synonymous single nucleotide polymorphisms (nsSNP), categorizing them on a scale from likely benign to likely harmful. This tool broadens the Grantham difference scope to include multiple comparisons and MSA. By entering amino acid sequences and mutations, it provides classifications from class 15, deemed neutral, to class 65, regarded as deleterious.^23^ Mutants that were identified as deleterious, impacting stability and altering biophysical properties, were further assessed for the degree of evolutionary conservation using the ConSurf web server.^22^

### SNP effect

The characterization process involved utilizing the human SNP effect server. This server is primarily designed for phenotyping analysis and plays a crucial role in annotating variants within the human proteome and providing molecular-level characterization of diseases and polymorphisms. To enhance its predictive capabilities, the server incorporates four powerful tools: TANGO, WALTZ, LIMBO and FoldX. TANGO is employed to identify protein regions susceptible to aggregation, while WALTZ is used to discern phylogenetic regions. Additionally, LIMBO is utilized to predict regions with an affinity for binding to the hsp70 chaperone. Furthermore, FoldX plays a crucial role in assessing the impact of mutations on structural stability. By incorporating these tools, the SNP effect server ensures a comprehensive and detailed analysis of SNP effects at a molecular level.

## Results

### Clinical manifestations and biochemical profiles in patients with MSUD

The study encompassed a total of 20 MSUD patients from the Children’s Hospital Zhejiang University School of Medicine (Zhejiang, China), consisting of twelve males and eight females, who were diagnosed with an average/median age of 14.80 ± 5.44 days. All patients had a regular birth history, and the mean birth weight was 3241.05 ± 428.43 g. In the cohort, the diagnosis was detected by MS/MS during newborn screening; 19 patients were diagnosed during the neonatal period in the meantime with clinical symptoms, and one patient had no clinical findings and was diagnosed at 31 days with abnormal MS/MS results. The majority of the patients primarily exhibited symptoms of poor feeding and lethargy within a few days after birth, and a subset of these patients also experienced seizures. Except for six patients whose initial age of clinical presentation was unclear, the average age of onset for the remaining individuals was determined to be 7.86 days, with a standard deviation of ±5.14 days.

**Figure 1.**
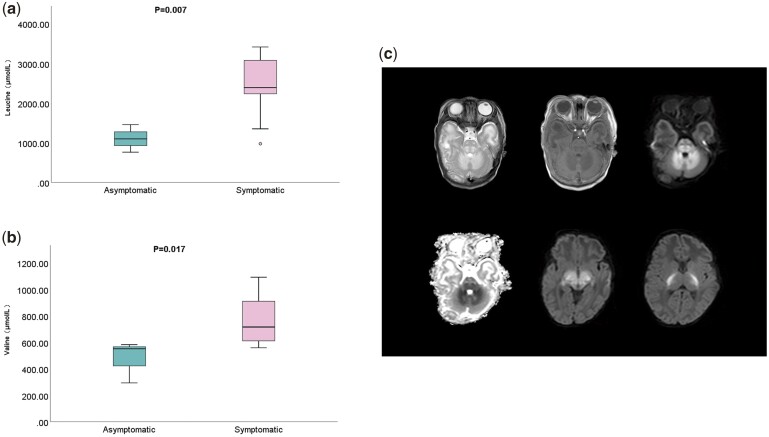
Correlation of amino acid levels and neuroimaging findings in MSUD. The concentrations of leucine (**a**) and valine (**b**) were significantly correlated with the clinical manifestations of MSUD patients, *P* values of less than 0.05 were considered as statistically significant. The bilateral dentate nuclei of the cerebellum and pons showed symmetrical, abnormally changed signal intensity (**c**). T2-weighted showed elevated signal intensity, T1-weighted showed decreased signal intensity, diffusion-weighted and ADC showed diffusion restriction. The diffusion-weighted of the bilateral thalamus and the posterior limb of the bilateral internal capsule showed symmetrical diffusion restriction.

All of the patients’ MS/MS revealed significantly elevated leucine and valine. The concentration of leucine is between 760.71 and 3411.81 μmol/L, while the concentration of valine is between 293.24 and 1091.62 μmol/l. There were significant differences in the attention of leucine and valine in NBS results between symptomatic and asymptomatic MSUD patients (Figure 1a and b).

All the patients were called when the newborn screening results came in. Except for three patients whose onset time was unclear and three patients were asymptomatic at the time of confirmatory testing, 14 patients already had symptoms at the time of notification, including lethargy, poor feeding and even seizure. MRI was performed on 10 patients, all of whom had abnormal signal changes in the brain (Figure 1c). Unexpectedly, we found that six of these children had congenital heart disease. Despite treatment being given timely, 11 patients died due to illness, mainly in the neonatal period. Of the nine children who survived, all were treated with a specialist metabolic dietitian, and three had liver transplantations. There were differences in clinical manifestations during the survivors' follow-up. Except for two infants who are still under observation at present, five patients have mental retardation, while two patients have no apparent abnormalities (Table 1).

**Table 1.** Patient information

No.	Gender	Gestational age (week)	Birth weight (g)	Age of diagnosis (day)	Clinical symptoms	Age of onset (day)	Congenital heart disease	Outcome (time of death)	Liver transplantation	Leucine (μmol/L)	Valine (μmol/L)
1	F	37	2700	21	Asymptomatic	–	No	Death (10 y)	No	1094.69	293.24
2	M	39	2650	11	Poor feeding, seizure	1	Yes	Death (15d)	No	2832.99	629.98
3	M	40	3830	16	–	–	No	Death (1m4d）	No	973.01	569.13
4	F	37	2950	12	Poor feeding, seizure	6	No	Death (27d）	No	1393.19	591.90
5	M	40	–	10	Seizure	–	No	Death	No	3089.10	664.49
6	M	38	2700	23	Poor feeding, lethargy, seizure	6	Yes	Mental retardation	No	3096.72	802.12
7	F	39	3100	54	Asymptomatic	–	No	Normal	No	760.71	582.28
8	M	39	3200	12	Poor feeding, lethargy, seizure	4	No	Death (19d)	No	3411.81	600.20
9	M	38	3600	16	Poor feeding, lethargy, seizure	4	Yes	Death (18d）	No	1679.73	756.23
10	F	39	3200	12	–	–	No	Death	No	3072.86	979.29
11	M	38	3200	22	Asymptomatic	19	No	Mental retardation	No	1454.03	550.76
12	F	40	3130	8	Poor feeding	6	Yes	Normal	Yes	3165.42	558.28
13	M	39	3780	7	–	–	No	Death (15d）	No	2252.81	632.69
14	F	38	3050	20	Poor feeding, seizure	18	No	Mental retardation	No	2233.56	715.59
15	M	39	4150	12	Poor feeding, seizure	5	No	Mental retardation	Yes	2562.69	914.15
16	M	37	2900	13	Poor feeding, lethargy, seizure	7	No	Death (13d)	No	2387.29	909.44
17	F	38	3150	11	Poor feeding, lethargy, seizure	11	No	Mental retardation	Yes	1349.07	610.94
18	F	38	3300	14	Poor feeding, lethargy, seizure	9	Yes	Follow-up	No	2355.60	1091.62
19	M	38	3070	9	Poor feeding, seizure	5	Yes	Follow-up	No	2427.70	1025.15
20	M	38	3920	13	Poor feeding, lethargy	10	No	Death (1m4d)	No	2360.82	909.62

y, year; m, month; d, day; –, unknown.

### Genotypic diversity and mutation spectrum in MSUD patients

**Figure 2.**
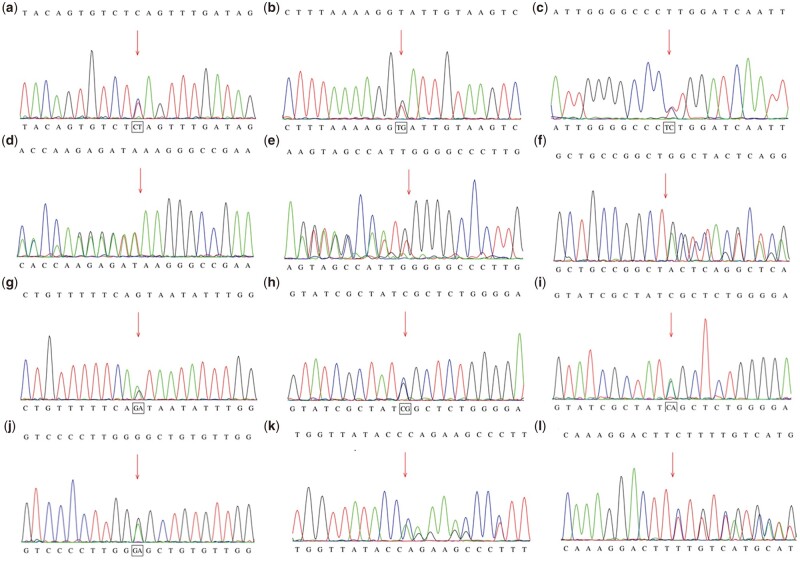
Twelve novel variants were detected. (**a**) c.280C>T in *DBT* gene; (**b**) c.433 + 2T>G in *DBT* gene; (**c**) c.1268T>C in *DBT* gene; (**d**) c.500del in *DBT* gene; (**e**) c.1264dup in *DBT* gene; (**f**) c.29_32del in *BCKDHB* gene; (**g**) c.275-1G>A in *BCKDHB* gene; (**h**) c.508C>G in *BCKDHB* gene; (**i**) c.508C>A in *BCKDHB* gene; (**j**) c.559G>A in *BCKDHB* gene; (**k**) c.645del in *BCKDHB* gene; (**l**) c.673_675del in *BCKDHB* gene.

Fifteen patients underwent genetic testing. All of them had compound heterozygous variants. The *BCKDHB* gene was the most commonly affected (53.3%) compared to the *BCKDHA* gene (20.0%) and *DBT* gene (26.7%). Three patients harbored variants in the *BCKDHA* gene, eight had variants in the *BCKDHB* gene and four carried variants in the *DBT* gene. A total of 25 different variants were detected, including 13 missense variants (52%), six frameshift variants, three nonsense variants, two splice site variants and one small deletion variant (Table 2). According to the ExAC and gnomAD databases, none of these variants have been described in the general population in the homozygous state. Twelve variants were novel (c.280C>T, c.433 + 2T>G, c.500del, c.1264dup, c.1268T>C in the *DBT* gene and c.29_32del, c.275-1G>A, c.508C>G, c.508C>A, c.559G>A, c.645del, c.673_675del in the *BCKDHB* gene). c.1046G>A (3/13) and c.853C>T (2/13) were the most prevalent variants in the *BCKDHB* gene, while c.1310_1311del (2/5) and c.75_76del (2/7) appeared most frequently in *BCKDHA* gene and *DBT* gene, respectively (Figure 2).

**Table 2.** Mutations detected in 15 Chinese patients with MSUD

Patient ID	Gene	Transcript	Variants	Resource	ACMG classification*
5	*BCKDHB*	NM_183050.4	c.1036A>G (p.Q346R)	Maternal	VUS
			c.1046G>A (p.C349Y)	Paternal	LP
6	*BCKDHB*	NM_183050.4	c.410C>T (p.A137V)	Maternal	P
			c.1046G>A (p.C349Y)	Paternal	LP
7	*DBT*	NM_001918.5	c.433 + 2T>G	Paternal	LP
			c.1268T>C (p.L423P)	Maternal	VUS
9	*BCKDHB*	NM_183050.4	c.673_675del (p.L226del)	Paternal	VUS
			c.853C>T (p.R285*)	Maternal	P
10	*DBT*	NM_001918.5	c.252G>T (p.W84C)	Paternal	LP
			c.75_76del (p.C26Wfs*2)	Maternal	P
11	*BCKDHB*	NM_183050.4	c.508C>G (p.R170G)	Maternal	VUS
			c.767A>G (p.Y256C)	Paternal	VUS
12	*BCKDHB*	NM_183050.4	c.29_32del (p.W10Yfs*61)	Paternal	P
			c.275-1G>A	Maternal	P
13	*BCKDHA*	NM_000709.4	c.632C>T (p.T211M)	Paternal Maternal	LP
			c.1310_1311del (p.H437Lfs*8)		LP
14	*BCKDHB*	NM_183050.4	c.559G>A (p.G187S)	Paternal	VUS
			c.1046G>A (p.C349Y)	Maternal	LP
15	*BCKDHA*	NM_000709.4	c.712G>A (p.E238K)	Paternal	VUS
			c.1310_1311del (p.H437Lfs*8)	Maternal	LP
16	*DBT*	NM_001918.5	c.280C>T (p.Q94*)	Paternal	P
			c.75_76del (p.C26Wfs*2)	Maternal	P
17	*DBT*	NM_001918.5	c.500del (p.K167Rfs*22)	Paternal	P
			c.1264dup (p.A422Gfs*6)	Maternal	P
18	*BCKDHA*	NM_000709.4	c.308T>C (p.L103P)	Paternal	VUS
			c.1279C>G (p.L427V)	Maternal	VUS
19	*BCKDHB*	NM_183050.4	c.508C>A (p.R170S)	Paternal	LP
			c.1159C>T (p.R387*)	Maternal	P
20	*BCKDHB*	NM_183050.4	c.645del (p.R216Efs*14)	Maternal	P
			c.853C>T (p.R285*)	Paternal	P

P, pathogenic; LP, likely pathogenic; VUS, variant of unknown significance.

*Here represents for stop codon in the description of a mutation.

### Pathogenicity and stability prediction MSUD-associated mutations

A total of 13 missense mutations were classified as either deleterious or neutral by PredictSNP software. Among the individual algorithms used by PredictSNP, MAPP, PhD-SNP, PolyPhen-1, PolyPhen-2, SIFT, SNAP and PANTHER identified 11, 10, 12, 11, 11, 12, 10 and 10 mutations as deleterious, respectively (Table 3). The iStable server integrated the predictions from iMutant, MUpro and iStable. Specifically, iMutant, MUpro and iStable predicted 7, 10 and 10 mutations, respectively, that would reduce protein stability (Table 4).

**Table 3.** Deleteriousness prediction of *BCKDHB, BCKDHA* and *DBT* mutations using the PredictSNP server

Accession number	Amino acid change	Predict SNP	MAPP	PhD-SNP	PolyPhen-1	PolyPhen-2	SIFT	SNAP	PANTHER
NP_898871.1	Q346R	D	D	D	D	D	D	N	N
NP_898871.1	C349Y	D	D	D	D	D	D	D	D
NP_898871.1	A137V	D	D	D	D	D	D	D	D
NP_898871.1	R170G	D	D	D	D	D	D	D	D
NP_898871.1	Y256C	D	N	D	D	D	D	D	D
NP_898871.1	G187S	N	N	D	N	D	N	N	N
NP_898871.1	R170S	D	D	D	D	D	D	D	D
QVG60139.1	T211M	D	D	D	D	D	D	D	D
QVG60139.1	E238K	D	D	D	D	D	D	D	D
QVG60139.1	L103P	D	D	D	D	D	D	D	D
QVG60139.1	L427V	N	N	N	N	N	D	N	N
NP_001909.4	L423P	D	D	D	D	N	D	D	D
NP_001909.4	W84C	D	D	D	D	D	D	D	D

D, deleterious; N, neutral; U, unknown.

**Table 4.** Change in stability prediction of *BCKDHB, BCKDHA* and *DBT* mutations using the iStable server

Accession number	Amino acid change	i-Mutant2.0 SEQ	DDG	MUpro	Conf.score	iStable	Conf.score
NP_898871.1	Q346R	I	0.23	I	0.25948117	I	0.766507
NP_898871.1	C349Y	I	−0.35	D	−0.022997033	D	0.676555
NP_898871.1	A137V	I	0.52	I	0.29907719	I	0.803662
NP_898871.1	R170G	D	−1.73	D	Null	D	0.675816
NP_898871.1	Y256C	I	−0.43	D	−0.70581171	D	0.581346
QVG60139.1	T211M	I	0.08	I	0.0097364644	I	0.837078
NP_898871.1	G187S	D	−1.23	D	−0.72686453	D	0.72686453
NP_898871.1	R170S	D	−1.54	D	Null	D	0.657017
QVG60139.1	E238K	D	−0.68	D	−0.9406555	D	0.803146
QVG60139.1	L103P	I	−1.16	D	−1	D	0.774927
QVG60139.1	L427V	D	−1.24	D	−0.20493536	D	0.832677
NP_001909.4	L423P	D	−1.73	D	−0.90249807	D	0.768657
NP_001909.4	W84C	D	−1.32	D	−1	D	0.760846

D, decrease; I, increase.

### Biophysical impact and evolutionary conservation of selected MSUD mutations

**Figure 3.**
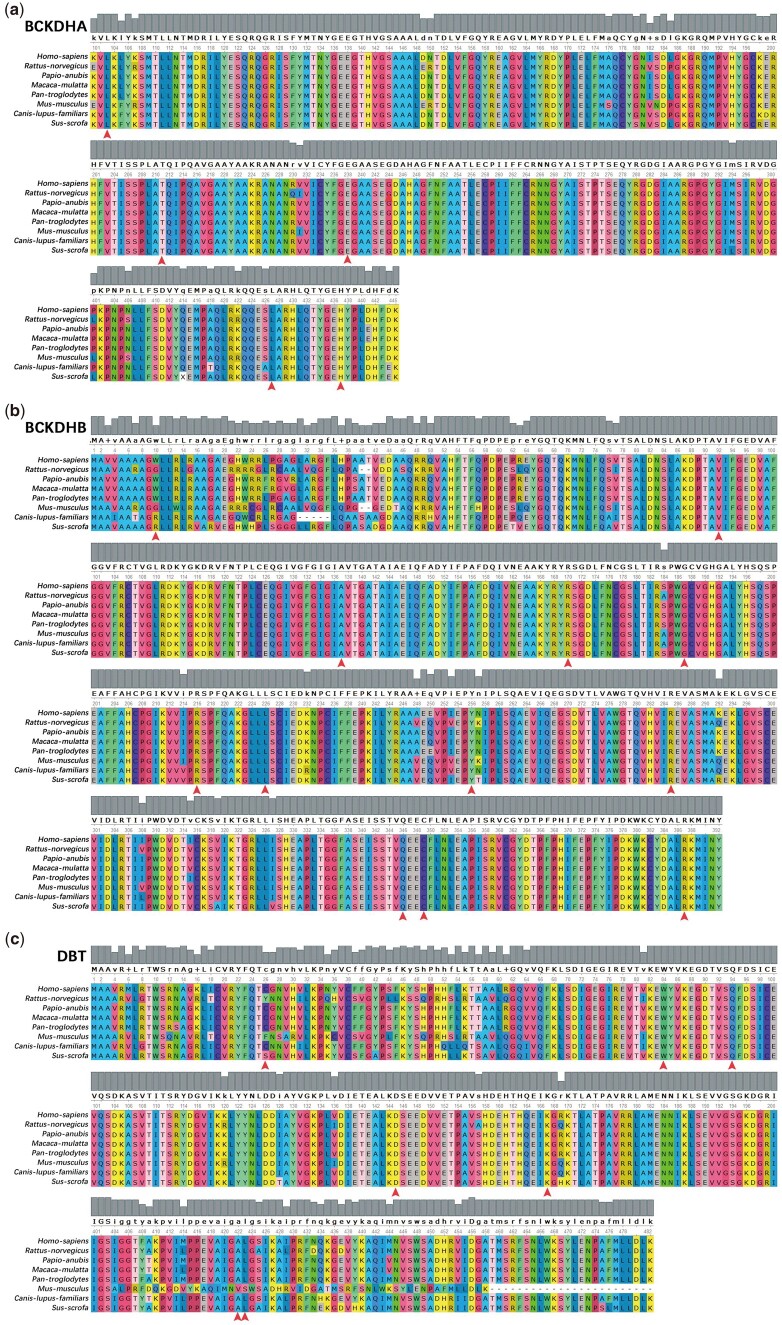
Comparative analysis of mutations in MSUD-related genes. Conservation analysis of pathogenic mutations amino acid positions of the *BCKDHA* (**a**), *BCKDHB* (**b**) and *DBT* (**c**) using the UGENE.

The Align GVGD tools were employed to investigate changes in the biophysical properties of amino acids. Among the 13 mutations examined, eight were classified as class C65, indicating a higher likelihood of causing damage (Table 5). Taking into account the results obtained from all the tools, five specific mutants, namely E238K (BCKDHA), R170G (BCKDHB), R170S (BCKDHB), L423P (DBT) and W84C (DBT), were selected for further analysis (Table 6). The aforementioned computational tools uniformly predicted these mutants as deleterious, indicating a potential reduction in protein stability (Tables 2–4). Subsequently, each identified mutation was analyzed using the UGENE software to assess their evolutionary conservation. Most of them demonstrated a high degree of conservation (Figure 3).

**Table 5.** Biophysical characterization of *BCKDHB, BCKDHA* and *DBT* mutations using the Align GVGD server

Accession number	Amino acid change	GV	GD	Prediction
NP_898871.1	Q346R	0.00	42.81	Class C35
NP_898871.1	C349Y	0.00	193.72	Class C65
NP_898871.1	A137V	0.00	64.43	Class C55
NP_898871.1	R170G	0.00	125.13	Class C65
NP_898871.1	Y256C	0.00	193.72	Class C65
NP_898871.1	G187S	0.00	55.27	Class C55
NP_898871.1	R170S	0.00	109.21	Class C65
QVG60139.1	T211M	0.00	81.04	Class C65
QVG60139.1	E238K	0.00	56.87	Class C55
QVG60139.1	L103P	0.00	97.78	Class C65
QVG60139.1	L427V	0.00	31.78	Class C25
NP_001909.4	L423P	0.00	97.78	Class C65
NP_001909.4	W84C	0.00	214.36	Class C65

QVG60139.1, *BCKDHA*; NP_898871.1, *BCKDHB*; NP_001909.4, *DBT*.

**Table 6.** Phenotypic effect prediction of pathogenic *BCKDHA, BCKDHB* and *DBT* mutations using the SNPeffect server

Accession number	Mutation	TANGO	WALTZ	LIMBO	FoldX
QVG60139.1	E238K	Decreases the aggregation tendency	Increases the amyloid propensity	Does not affect the chaperone binding tendency	Severely reduces the protein stability
NP_001909.4	L423P	Does not affect the aggregation tendency	Does not affect the amyloid propensity	Does not affect the chaperone binding tendency	Reduces the protein stability
NP_001909.4	W84C	Decreases the aggregation tendency	Does not affect the amyloid propensity	Does not affect the chaperone binding tendency	Could not find reliable structural information
NP_898871.1	R170G	Does not affect the aggregation tendency	Does not affect the amyloid propensity	Does not affect the chaperone binding tendency	Slightly reduces the protein stability
NP_898871.1	R170S	Does not affect the aggregation tendency	Does not affect the amyloid propensity	Does not affect the chaperone binding tendency	Slightly reduces the protein stability

QVG60139.1, *BCKDHA*; NP_898871.1, *BCKDHB*; NP_001909.4, *DBT*.

### *In silico* assessment of amino acid substitutions on protein stability and activity in MSUD

**Figure 4.**
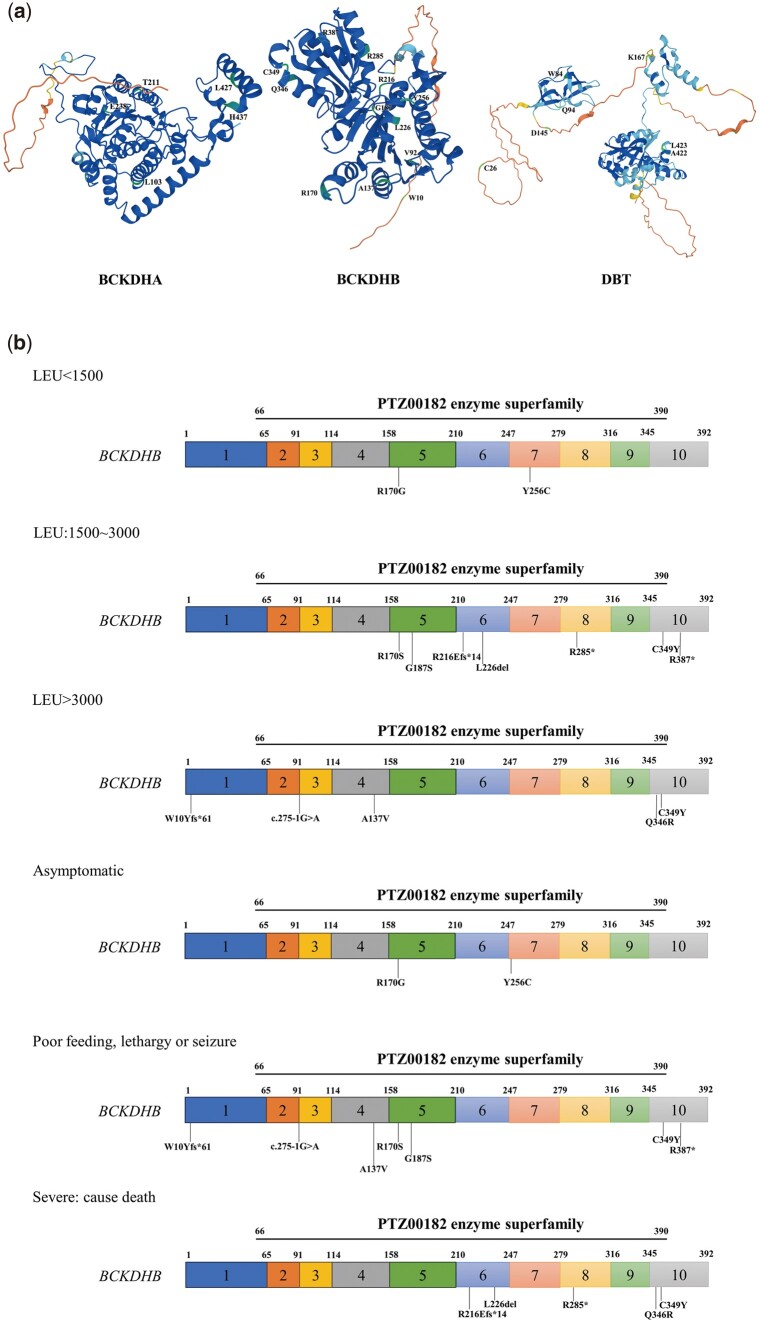
MSUD gene variants: *BCKDHA, BCKDHB, DBT* analysis and clinical correlations. The domain of the *BCKDHA, BCKDHB* and *DBT* (**a**). The relationships of the distribution of variants and phenotypes of patients (**b**). The distribution of variants in exons and domains based on three concentrations of leucine: (1) <1500; (2) 1500–3000; (3) >3000; The distribution of variants in exons and domains based on three clinical phenotypes: (1) asymptomatic; (2) intermediate phenotypes presented with poor feeding, lethargy or seizure may lead to mental retardation; (3) severe phenotypes lead to death.

The substitution of single amino acids has the potential to disrupt and alter protein stability. By analyzing the output generated from various *in silico* tools, mutational analysis was conducted, and mutations were introduced at the corresponding coordinates using Swiss-Pdb Viewer. The crystal structures of native human BCKDHA, BCKDHB and DBT proteins were obtained from the Protein Data Bank. Assessing the changes in physicochemical properties induced by mutations aids in understanding the impact of these mutations on protein activity. The results showed that T211 (BCKDHA), V92, A137, R170, L226, R285, R387, Q346 (BCKDHB), W84, A422, L423 (DBT) were located at ordered structure summarized below by Alphafold website (Figure 4a). We also outlined the relationship between the mutation domain and leucine concentration (Figure 4b). The variants were mainly distributed in the domain of PTZ00182 enzyme superfamily of BCKDHB. The distribution of variants in exons and domains based on three clinical phenotypes were also analyzed.

## Discussion

Most MSUD patients have the severe classic form, characterized by neonatal onset of encephalopathy and coma.^26^ According to the Human Gene Mutation Database (HGMD), over 300 variants of MSUD have been documented, with a majority being missense variants.^27^ However, a comprehensive genotype–phenotype relationship in MSUD has not been fully established. Our study sought to investigate the genetic mutations, clinical implications and correlation of MSUD in a large cohort of the Chinese population. 20 MSUD patients were identified from 4.8 million neonatal screenings, and 25 variants were detected (Tables 1 and 2). Most patients exhibited symptoms within the first 10 days of life, and the *BCKDHB* gene emerged as the primary causative gene in MSUD in our cohort, in accordance with the results of previous studies.^12^^,^^27^ Among these mutations, 12 novel variants were identified, which expands the mutation spectrum of MSUD. Moreover, our findings revealed a significantly lower incidence (1 in 244306) of MSUD in the Chinese population than the global average (1:84034).^5^ Specific environmental, genetic or dietary factors in the Chinese population may cause it. The study's outcomes are pivotal in comprehending regional variations in MSUD incidence and its genetic framework.

Fortunately, the routine inclusion of MSUD in neonatal screening programs in China likely contributes to relieving the phenotype of patients with MSUD by more objective early management.^27^ Given the rapid onset of symptoms in many patients and the available effective intervention strategies for MSUD, the challenges of MSUD are earlier screening protocols and prediction of the disease progression. Additionally, the significance of prenatal diagnosis in managing MSUD is underscored by identifying mutations associated with the disease, enabling at-risk families to make informed decisions.^11^^,^^28^ Therefore, establishing the genotype–phenotype relationship in MSUD is merit and urgent for developing personalized treatment strategies and predicting patient outcomes. Recent studies align with our findings on the impact of genetic variability on MSUD’s clinical presentations.^29^ A 2020 study further corroborates our observations of genotype–phenotype relationship, linking specific genetic mutations to clinical symptom severity.^30^ A 2021 study validates our approach, demonstrating the effectiveness of targeted NGS in the Chinese Han population for MSUD diagnosis, highlighting the importance of advanced genetic screening.^31^

In this study, the implementation of NGS and sophisticated bioinformatics pushed forward further. Beyond the undetected sequencing, six deaths contained one in *BCKDHA* (T211M/H437Lfs*8), three in *BCKDHB* (R216Efs*14/R285*, L226del/R285* and Q346R/C349Y) located in PTZ00182 enzyme superfamily, two in *DBT* (W84C/C26Wfs*2 and Q94*/C26Wfs*2). H437Lfs*8 in *BCKDHA* occurred in two people, and the remaining showed mental retardation even if he has had liver transplantation. C349Y in *BCKDHB* occurred in three people, and the remaining exhibited congenital heart disease/mental retardation and mental retardation. R285* in *BCKDHB* occurred in two dead people. C26Wfs*2 in *DBT* occurred in two dead people. Given together, R285* in *BCKDHB* and C26Wfs*2 in *DBT* were highly pathogenetic (Tables 1 and 2), along with their evolution conservation and functional domain (Figures 3 and 4). W10Yfs*61 (BCKDHB), L423P (DBT), and A422Gfs*6 (DBT) were not very conserved in evolution, and the suffered patients with normal phenotype or mental retardation (Table 1 and Figure 3). Beyond these, other mutations all showed heavy disease phenotypes. Together, mutation at conservation would lead to a more severe phenotype. Several software tools predicted *BCKDHA* (c.1279C>G (p.L427V)) and *BCKDHB* (c.559G>A (p.G187S)) as neutral, while *BCKDHB* (c.767A>G (p.Y256C)) and *BCKDHB* (c.1036A>G (p.Q346R)) were also predicted as neutral by a few software tools. Of note, the L427V mutation was derived from the 18th patient (who also harbored the high pathogenicity mutation c.308T>C (p.L103P) presented in Table 5), with an onset age of day 9. Similarly, the G187S mutation was derived from the 14th patient (who also carried the c.1046G>A (p.C349Y) mutation), with an onset age of day 18. The Y256C mutation was derived from the 11th patient (who also had the c.508C>G (p.R170G) mutation), with an onset age of day 19. For the Q346R mutation was derived from the 5th patient (who also carried the c.1046G>A (p.C349Y) mutation), the onset age was unknown and have dead, but the diagnosis age was day 10. Preliminarily, these mutations predicted as neutral indeed seem to exhibit a slightly later onset age. Additionally, we have included results from other prediction software, including the recently published MAGPIE,^25^ in Supplementary Tables S1 and S2. While MAGPIE also suggests these four variants as weakly pathogenic, some other software tools still consider them pathogenic. Additionally, our findings emphasize the importance of exploring the domain of the PTZ00182 enzyme superfamily of the BCKDHB gene concerning patient phenotypes and leucine concentrations, which could further refine diagnostic and treatment strategies.

Our research provides a more extensive analysis of MSUD within a Chinese cohort compared to prior studies. Furthermore, the discrepancy between the severity predicted by specific genotypes and the actual clinical presentations could be attributed to the early and effective intervention strategies implemented in China, highlighting the importance of prompt treatment in managing this condition.

Despite its strengths, our study has limitations. The sample size, while larger than previous research in China, is still relatively small compared to global studies, which may introduce bias and affect the precision of our findings.^32^^,^^33^ This limitation might explain the discrepancies between our results and those of other countries, such as the Mennonite community, where it is about 1 in 380 live births.^9^ In addition, timely intervention for MSUD upon NBS detection could have mitigated disease progression, which may affect genotype–phenotype relationship. The lack of comprehensive data on these factors, e.g. environmental and dietary factors, can limit the ability to fully understand the interplay between genetics and environment in disease manifestation.^34^ In line with current advancements, the management of MSUD is evolving. New therapeutic approaches are being investigated, including gene therapy and advanced dietary management strategies.^35^ These developments could offer more effective management strategies for MSUD, particularly for patients with severe genotypes.^29^ Further studies are needed to understand different mutations' phenotypic effects and develop more personalized treatment approaches.

## Supplementary Material

hcae104_Supplementary_Data
