# Supplementary material for: Genotypic and phenotypic spectrum of maple syrup urine disease in Zhejiang of China
Source: QJM. 2024 Jun 5;117(10):717–27. doi: 10.1093/qjmed/hcae104 (PMC11604211; doi:10.1093/qjmed/hcae104)
Supplement: hcae104_Supplementary_Data [file hcae104_supplementary_data.zip › hcae104_Supplementary_Data/Abbreviations.docx]

**Abbreviations:**

| ACMG | American College of Medical Genetics and Genomics |
| --- | --- |
| BCAAS | Branched-chain amino acids |
| BCDKH | Branched-chain α-ketoacid dehydrogenase |
| BWA | Burrows-Wheeler Aligner |
| ExAC | Exome Aggregation Consortium |
| MAF | Minor allele frequency |
| MSA | Multiple sequence alignment |
| MSUD | Maple syrup urine disease |
| NBS | Newborn screening |
| NGS | Next-generation sequencing |
| ROS | Reactive oxygen species |
| SNP | Single nucleotide polymorphism |
| SVM | Support vector machine |
