# Supplementary material for: Genotypic and phenotypic spectrum of maple syrup urine disease in Zhejiang of China
Source: QJM. 2024 Jun 5;117(10):717–27. doi: 10.1093/qjmed/hcae104 (PMC11604211; doi:10.1093/qjmed/hcae104)
Supplement: hcae104_Supplementary_Data [file hcae104_supplementary_data.zip › hcae104_Supplementary_Data/supplement table.docx]

Supplement Table 1. Predicting the pathogenicity of MUSD mutations using the MAGPIE website

| Gene | Amino acid change | Accession number | Chr | Start | End | MAGPIE prediction |
| --- | --- | --- | --- | --- | --- | --- |
| BCKDHA | L427V | QVG60139.1 c.1279C>G | 19 | 41424549 | 41424549 | 0.290306226 |
| BCKDHB | Y256C | NP_898871.1 c.767A>G | 6 | 80200958 | 80200958 | 0.749243776 |
| BCKDHB | G187S | NP_898871.1 c.559G>A | 6 | 80168957 | 80168957 | 0.963002921 |
| BCKDHB | Q346R | NP_898871.1 c.1036A>G | 6 | 80273220 | 80273220 | 0.889724628 |

MAGPIE output ranges from 0-1; 0 indicates a benign mutation, while 1 indicates a highly pathogenic mutation

Supplement Table 2. Predicting the pathogenicity of MUSD mutations using the website

| Gene | Amino acid change | LRT | | MutationTaster | FATHMM | MetaSVM | MetaLR |
| --- | --- | --- | --- | --- | --- | --- | --- |
| BCKDHA | L427V | | D | D | D | D | D |
| BCKDHB | Y256C | | D | D | D | D | D |
| BCKDHB | G187S | | D | D | D | D | D |
| BCKDHB | Q346R | | D | N | D | D | D |

D—Deleterious, N—Neutral
